# Alveolar recruitment can be predicted from airway pressure-lung volume loops: an experimental study in a porcine acute lung injury model

**DOI:** 10.1186/cc6771

**Published:** 2008-01-21

**Authors:** Jacob Koefoed-Nielsen, Niels Dahlsgaard Nielsen, Anders J Kjærgaard, Anders Larsson

**Affiliations:** 1 Department of Anesthesia and Intensive Care, Aarhus University Hospital, Aalborg, Hobrovej 18-22, DK-9000 Aalborg, Denmark; 2 Department of Anesthesia and Intensive Care, Aarhus University Hospital, Århus, Norrebrogade 44, DK-8000 Århus, Denmark

## Abstract

**Introduction:**

Simple methods to predict the effect of lung recruitment maneuvers (LRMs) in acute lung injury (ALI) and acute respiratory distress syndrome (ARDS) are lacking. It has previously been found that a static pressure–volume (PV) loop could indicate the increase in lung volume induced by positive end-expiratory pressure (PEEP) in ARDS. The purpose of this study was to test the hypothesis that in ALI (1) the difference in lung volume (Δ*V*) at a specific airway pressure (10 cmH_2_O was chosen in this test) obtained from the limbs of a PV loop agree with the increase in end-expiratory lung volume (ΔEELV) by an LRM at a specific PEEP (10 cmH_2_O), and (2) the maximal relative vertical (volume) difference between the limbs (maximal hysteresis/total lung capacity (MH/TLC)) could predict the changes in respiratory compliance (Crs), EELV and partial pressures of arterial O_2 _and CO_2 _(PaO_2 _and PaCO_2_, respectively) by an LRM.

**Methods:**

In eight ventilated pigs PV loops were obtained (1) before lung injury, (2) after lung injury induced by lung lavage, and (3) after additional injurious ventilation. Δ*V *and MH/TLC were determined from the PV loops. At all stages Crs, EELV, PaCO_2 _and PaO_2 _were registered at 0 cmH_2_O and at 10 cmH_2_O before and after LRM, and ΔEELV was calculated. Statistics: Wilcoxon's signed rank, Pearson's product moment correlation, Bland–Altman plot, and receiver operating characteristics curve. Medians and 25th and 75th centiles are reported.

**Results:**

Δ*V *was 270 (220, 320) ml and ΔEELV was 227 (177, 306) ml (*P *< 0.047). The bias was 39 ml and the limits of agreement were – 49 ml to +127 ml. The *R*^2 ^for relative changes in EELV, Crs, PaCO_2 _and PaO_2 _against MH/TLC were 0.55, 0.57, 0.36 and 0.05, respectively. The sensitivity and specificity for MH/TLC of 0.3 to predict improvement (>75th centile of what was found in uninjured lungs) were for EELV 1.0 and 0.85, Crs 0.88 and 1.0, PaCO_2 _0.78 and 0.60, and PaO_2 _1.0 and 0.69.

**Conclusion:**

A PV-loop-derived parameter, MH/TLC of 0.3, predicted changes in lung mechanics better than changes in gas exchange in this lung injury model.

## Introduction

Lung collapse is an important cause of deteriorated oxygenation and gas exchange after major surgery, in acute lung injury (ALI) and in acute respiratory distress syndrome (ARDS) [1,2]. Although the logical therapy for lung collapse, namely a lung recruitment maneuver (LRM) in combination with high positive end-expiratory pressure (PEEP), improves oxygenation in these conditions, it has not conclusively been found to improve important outcome measures, for example length of stay in the hospital or mortality [3-6].The reasons for the latter might be that in the studies the positive effects of LRM in patients with recruitable lung collapse are evened out by the negative effects such as circulatory compromise and barotrauma/volutrauma in non-recruiters. This indicates that LRM preferably should be performed only in patients with lung collapse that it is possible to recruit [7,8]. Although examination of the lungs by computed tomography could assess the effect of LRMs, it is complicated and the patient will be exposed to radiation and needs to be moved to the computed tomography suite [9,10]. Therefore an easy method for predicting the effect of LRMs would be useful.

Superimposed plots of inspiratory airway pressure against lung volume (pressure–volume; PV) obtained from different PEEP levels were originally described by Ranieri and coworkers, and have been further developed by others, for assessing PEEP-induced lung recruitment [11,12]. However, this method does not predict whether an LRM would be successful, but instead shows the volume effect of derecruitment caused by removal or reduction of PEEP [13]. Vieillard-Baron and coworkers proposed a slow inflation–deflation (upper airway pressure of 20 cmH_2_O) PV loop method for predicting the volume effect by PEEP-induced lung recruitment [14]. They found in ARDS that the increase in lung volume, from zero end-expiratory pressure (ZEEP) to the airway pressure equal to the subsequent PEEP, assessed from the difference between the expiratory and inspiratory limbs of the loop, agreed well with decrease in volume found at removal of PEEP. In addition, they found in patients with lower inflexion points at high pressures that PEEP recruited more lung volume than it did in patients without any obvious lower inflexion points. We hypothesized that a modification of this method, by measuring end-expiratory lung volume (EELV), using higher airway pressures (which is commonly used in LRM) and measuring the volume difference between the limbs of the PV loop (hysteresis), might predict the effects of a subsequent LRM (evaluated by changes in EELV, oxygenation, compliance of the respiratory system (Crs) and CO_2 _elimination).

In ALI/ARDS, the inspiratory limb reflects mainly lung recruitment and the expiratory limb reflects derecruitment [15,16]. At a specific pressure, the volume hysteresis reflects the volume recruited (and the expansion of the recruited volume) by the PV-loop maneuver. Thus, a substantial hysteresis would predict that an LRM would be effective, whereas a minor hysteresis would indicate that an LRM would not be beneficial.

**Figure 1:**
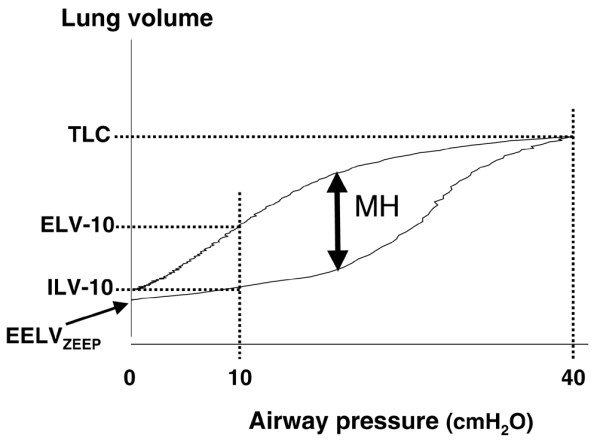
An airway pressure – absolute lung volume loop from an animal after lung lavage. EELV_ZEEP_, end-expiratory lung volume at zero end-expiratory airway pressure; ILV-10 and ELV-10, absolute lung volumes at an airway pressure of 10 cmH_2_O obtained from the inspiratory limb and from the expiratory limb, respectively; TLC, total lung capacity; MH, maximal volume hysteresis.

The aim of the present study was to test this hypothesis in a porcine model with normal lungs, lungs subjected to lavage and finally lungs subjected to lavage and injurious ventilation (1) by registering PV loops and volume hysteresis under the three conditions and then compare hysteresis (assumed predicted recruited lung volume) at 10 cmH_2_O airway pressure with the measured difference in EELV at 10 cmH_2_O PEEP before and after an LRM (the recruited volume plus expansion of recruited lung units), (2) to relate the maximal volume hysteresis (MH) on the PV curve standardized to total lung capacity (TLC) to changes in EELV, Crs and blood gases caused by an LRM (Figure 1), and (3) to calculate the sensitivity and specificity of using the MH/TLC ratio for predicting the effect of an LRM.

We found that the volume hysteresis at 10 cmH_2_O agreed with the increase in EELV, that MH/TLC was related to changes in EELV, Crs and PaCO_2_, and that a MH/TLC ratio of 0.3 predicted with high sensitivity and specificity whether an LRM would improve EELV, Crs, partial pressure of arterial CO_2 _(PaCO_2_) and partial pressure of arterial oxygen (PaO_2_).

## Materials and methods

This animal interventional study was performed at the laboratory of the Clinical Institute, Aarhus University Hospital. The study was approved by the Danish National Animal Ethics Committee.

### Anesthesia, ventilation and fluid management

Eight pigs, weighing 18 to 22 kg, were premedicated with midazolam 10 mg intramuscularly (i.m.), azaperone 80 mg i.m., and atropine 1 mg i.m. Anesthesia was induced with ketamine 2 mg/kg intravenously (i.v.) and fentanyl 5 μg/kg i.v. and maintained with ketamine 10 mg/kg per hour, fentanyl 5 μg/kg per hour, propofol 2 mg/kg per hour, and pancuronium 0.25 mg/kg per hour. The trachea was intubated (Portex tube, internal diameter 5.5 mm; Smiths Medical, London, UK), and the lungs were volume-controlled ventilated with a Servo 900C (Siemens-Elema, Solna, Sweden) with tidal volume 8 ml/kg, inspiratory/expiratory ratio 1:1, initial respiratory rate 12 breaths/min (adjusted before the main experiment to 20 to 30 breaths/min to achieve an arterial pH of about 7.4), and fraction of inspired oxygen 1.0. PEEP was initially set at 5 cmH_2_O. The dead space of the apparatus was 14 ml. Ringer acetate (20 ml/kg) was infused during the first hour and 10 ml/kg per hour for the rest of the experiment. Before the main experiment was initiated, 20 to 30 ml/kg Voluven (Fresenius Kabi, Uppsala, Sweden) was administered. Body temperature was maintained at 37 to 38°C.

At the end of the experiment the animals were killed with an intravenous overdose of pentobarbital.

### Instrumentation and measurement of arterial blood pressure and blood gases

A catheter was placed in the right common carotid artery for continuous monitoring of mean arterial blood pressure and for sampling of blood for analysis of PaO_2_, PaCO_2 _and pH (ABL 710; Radiometer, Copenhagen, Denmark). A central venous catheter was placed in the right internal jugular vein. A bladder catheter was inserted suprapubically to monitor urine flow.

### Measurements of lung volume and mechanics of the respiratory system

EELV was measured with an inert tracer gas washout technique by using sulfur hexafluoride [17,18].

Crs was calculated as Tidal volume/(End-inspiratory pressure – End-expiratory pressure). End-inspiratory and end-expiratory pressures were obtained after closure of the inspiratory and expiratory valves of the ventilator (pressing the hold-button of the ventilator) for 3 to 5 seconds.

PV loops from 0 to 40 cmH_2_O and back to 0 cmH_2_O were obtained by a slow inflation–deflation, interrupted technique, as reported previously [19]. In short, the lungs were slowly (60 ml/s) inflated via an interrupter from 0 to 40 cmH_2_O airway pressure. The pressure was kept constant at 40 cmH_2_O for 1 s, and then the lungs were passively deflated to 0 cmH_2_O via the interrupter, against a resistance. The interrupter worked in cycles of 320 ms with 160 ms opening and 160 ms occlusion. Airway pressure was measured (SCX01DN; Sensym, Rugby, UK) proximal to the interrupter and close to the endotracheal tube, between 80 and 150 ms after the start of each occlusion (that is, at zero flow and a stable pressure level), and the increment or decrement in volume was obtained by integration of the flow from mid-occlusion to mid-occlusion measured by a pneumotachograph (Gould 1; Fleish, Lausanne, Switzerland) placed distal to the interrupter. The pressure and volume signals were obtained at 200 Hz and were transmitted to a personal computer, which constructed the PV loops. The duration of the procedure was less than 1 minute. The PV loop was adjusted to absolute lung volume by adding the EELV at ZEEP (EELV_ZEEP_) to the registered volumes. From this loop the absolute lung volumes at an airway pressure of 10 cmH_2_O were obtained from the inspiratory limb (ILV-10) and from the expiratory limb (ELV-10) (Figure 1). MH was defined as the maximal difference in volume between the two limbs of the PV loop (Figure 1) [19]. TLC was defined as the lung volume at 40 cmH_2_O airway pressure (Figure 1). The figure of 40 cmH_2_O was chosen because it is usually a safe airway pressure and in animals with normal chest wall elastance, as in this experiment, it should generate an adequate transpulmonary pressure for obtaining accurate TLC also after lung injury.

### Induction of lung injury

Each animal was subjected to two kinds of lung injury: first, lung collapse produced by surfactant depletion by lung lavage, and second, mechanical lung injury by additional injurious ventilation of the surfactant-depleted lung. Lung lavage was performed at least 10 times with 20 ml/kg of normal saline at 37°C poured into the tracheal tube and removed by gravity or until no foam was observed in the removed fluid. The mechanical lung injury was achieved by ventilating the lungs for 30 minutes with peak airway pressures of 45 mmH_2_O, ZEEP, and a respiratory rate of 15/min. The instrumental dead space was increased during this procedure to avoid hypocapnia. After the procedure, the preceding ventilator settings were used.

### Experimental protocol and calculations

The pigs were placed in the supine position during the experiment. A PV loop was registered at the following times: (1) at baseline before induction of lung injury, (2) 30 minutes after lung lavage, and (3) 10 minutes after the end of the injurious ventilation. At each stage, EELV was measured at ZEEP (EELV_ZEEP_) and at 10 cmH_2_O PEEP before an LRM (EELV-10_noLRM_) and after an LRM (EELV-10_LRM_). At similar times Crs, PaCO_2 _and PaO_2 _were obtained. A prolonged end-expiratory hold was done before each measurement to insure that no intrinsic PEEP occurred. EELV_ZEEP _was measured after 5 minutes of ventilation at ZEEP. To ensure that the lungs were not inadvertently recruited before the measurement of EELV-10_noLRM_, the lungs were ventilated at ZEEP for 2 minutes before PEEP was set to 10 cmH_2_O, and the measurements were then made after 5 minutes. To prevent tidal lung recruitment, low inspiratory airway pressures (less than 22 cmH_2_O) were used. The LRM consisted of 2 minutes of pressure-controlled ventilation with a peak airway pressure of 40 cmH_2_O, PEEP 10 cmH_2_O, an inspiratory/expiratory ratio of 1:1 and a respiratory rate of 6 breaths/min. EELV-10_LRM _was measured 5 minutes after the LRM.

EELV_ZEEP _was used to adjust the PV loop to absolute lung volumes. The difference between EELV-10_LRM _and EELV-10_noLRM _(ΔEELV), which indicates the lung volume recruited plus the expansion of the recruited lung units at 10 cmH_2_O of PEEP, was compared with Δ*V*, defined as the difference between ELV-10 (the absolute lung volumes at an airway pressure of 10 cmH_2_O obtained from the expiratory limb of a static airway pressure – lung volume loop) and ILV-10 (the absolute lung volumes at an airway pressure of 10 cmH_2_O obtained from the inspiratory limb of an airway pressure – lung volume loop). Furthermore, MH found on the PV curve was standardized to TLC (MH/TLC) and related to the relative differences in EELV, Crs, PaCO_2 _and PaO_2 _between ventilation after and before LRM at a 10 cmH_2_O PEEP.

For the estimation of sensitivity and specificity of MH/TLC to predict the effect of a subsequent LRM, we considered an 'improvement' outside the interquartile centiles found before lung lavage as relevant.

### Statistics

All values are reported as medians and 25th and 75th centiles unless otherwise indicated.

Comparisons between and within the three lung conditions were analyzed with the Wilcoxon signed rank test. Data are not corrected for multiple comparisons. Each value was used for one or two comparisons. Regression analysis was performed by Pearson's product moment correlation. A Bland–Altman plot was used to analyze the agreement between ΔEELV and Δ*V *[20]. Analyses of receiver operating characteristics curves were used to determine the sensitivity and specificity of MH/TLC in predicting improvements in EELV, Crs, PaO_2 _and PaCO_2 _of an LRM. We considered *P *< 0.05 to be statically significant. The STATA software (StataCorp, College Station, TX, USA) was used for statistical analyses.

## Results

### Effect of lung lavage and injurious ventilation

**Figure 2:**
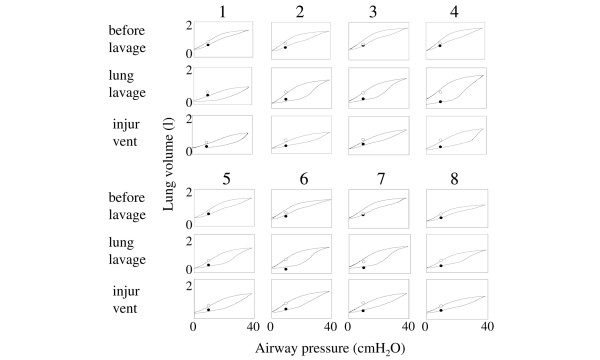
Static pressure–volume (PV) loops obtained in the eight animals under three lung conditions. The three conditions used were: before lung lavage, after lung lavage, and after lung lavage and additional injurious ventilation (injur vent). Each PV loop was obtained from 0 to 40 cmH_2_O and back to 0 cmH_2_O airway pressure by a slow inflation–deflation, interrupted technique. End-expiratory lung volume at 10 cmH_2_O of positive end-expiratory pressure before a lung recruitment maneuver (LRM) (EELV-10_noLRM_)(filled circles) and after an LRM (EELV-10_LRM_) (open circles) agreed well with the volumes found on the inspiratory and expiratory limbs, respectively, of the PV loops.

In comparison with baseline, EELV, Crs, PaO_2 _were decreased and PaCO_2 _was increased after lung lavage as well as after lung lavage and injurious ventilation (Table 1). These changes were mirrored in marked changes in the shapes of the PV loops from crescent to convex forms, increased hysteresis and rightward shifts of the lower inflexion points (Figure 2).

**Table 1:** Lung mechanics and blood gas tensions obtained at 10 cmH_2_O before and after LRM

Parameter	Before lung lavage		After lung lavage		After lung lavage and additional injurious ventilation	
	Before LRM	After LRM	Before LRM	After LRM	Before LRM	After LRM
EELV, l	0.68 (0.61, 0.71)	0.83^a ^(0.77, 0.86)	0.37^b ^(0.31, 0.46)	0.69^a ^(0.62, 0.78)	0.42^b ^(0.40, 0.46)	0.73^a ^(0.65, 0.78)
Crs, ml/cmH_2_O	9.5 (9.3, 10.1)	11.5^a ^(11.0, 12.0)	5.8^b ^(5.2, 6.6)	10.2^a ^(9.8, 11.0)	6.6^b ^(5.8, 7.0)	10.5^a ^(10.1, 10.8)
PaO_2_, kPa	71.2 (66.6, 80.0)	80.1^a ^(68.4, 82.3)	51.0^b ^(41.4, 56.4)	69.9^a ^(66.5, 77.7)	32.4^b ^(16.1, 45.6)	71.9^a ^(66.4, 76.2)
PaCO_2_, kPa	4.5 (4.3, 4.6)	4.4 (3.8, 5.0)	7.8^b ^(7.2, 9.7)	5.9^a ^(5.3, 7.2)	6.8^b ^(6.3, 7.4)	5.5^a ^(4.8, 6.3)

LRM, lung recruitment maneuver; PEEP, positive end-expiratory pressure; EELV, end-expiratory lung volume; Crs, compliance of the respiratory system; PaCO_2_, partial pressure of arterial CO_2_; PaO_2_, partial pressure of arterial oxygen.

The three lung conditions: before lung lavage, after lung lavage and after lung lavage and additional injurious mechanical ventilation

Results are presented as medians and 25th and 75th centiles.

^a^*P *< 0.05, before LRM compared with after LRM in the three lung conditions; ^b^*P *< 0.05, before lung lavage compared with after lung lavage or after lung lavage and additional injurious ventilation before the LRM.

### Effect of lung recruitment maneuver

EELV, Crs and PaO_2 _were increased at all lung conditions by the LRM (Table 1). However, PaCO_2 _decreased by the LRM only after lung lavage and after lung lavage and injurious ventilation.

### Comparisons between measured lung volumes before and after the lung recruitment maneuver and lung volumes obtained from the pressure–volume loops

Figure 2 shows that the measured lung volumes agreed well with the volumes found on the PV loops (EELV-10_noLRM _and ILV-10 were 464 ml (396, 615) and 417 ml (350, 665), respectively (*P *= 0.37), and EELV-10_LRM _and ELV-10 were 764 (665, 807) ml and 745 (640, 940) ml, respectively (*P *= 0.25). However, the volume gain predicted from the PV loops gave a systematic, minor overestimation as indicated by a Δ*V *of 270 (220, 320) ml compared with a ΔEELV of 227 (177, 306) ml (*P *< 0.047), and a bias (using Δ*V *and ΔEELV) of 39 ml. The limits of agreement were – 49 ml to +127 ml.

### MH/TLC versus relative changes in EELV, Crs, PaCO_2 _and PaO_2 _caused by the lung recruitment maneuver

**Figure 3:**
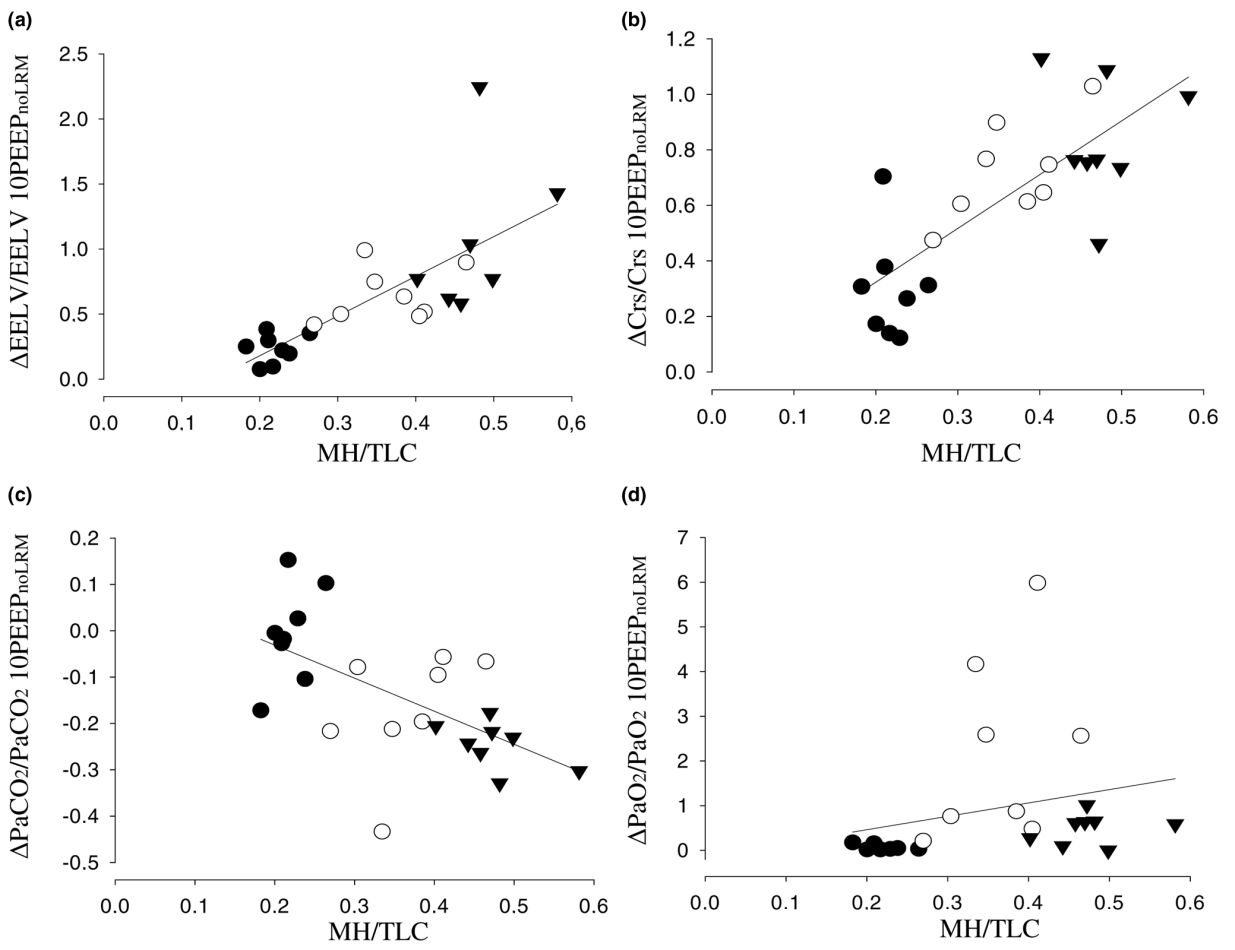
Relation between MH/TLC and lung mechanics or blood gas tensions. **(a) **Relation between the ratio between maximal volume hysteresis and total lung capacity (MH/TLC) and the relative changes at 10 cmH_2_O of positive end-expiratory pressure (PEEP) in EELV, **(b) **respiratory compliance, **(c) **partial pressure of arterial CO_2 _(PaCO_2_), and **(d) **partial pressure of arterial oxygen (PaO_2_) by a lung recruitment maneuver (LRM) in the three lung models. The regression lines are shown. The symbols depict the individual animals: filled circles, before lung lavage; open circles, after lung lavage; filled triangles, after lung lavage and additional injurious ventilation. ΔEELV/EELV 10PEEP_noLRM_, the ratio between the change in end-expiratory lung volume associated with LRM and the end-expiratory lung volume at 10 cmH_2_O PEEP before LRM; ΔCrs/Crs 10PEEP_noLRM_, the ratio between the change in compliance of the respiratory system associated with LRM and the compliance of the respiratory system at 10 cmH_2_O PEEP before an LRM; ΔPaCO_2_/PaCO_2 _10PEEP_noLRM_, the ratio between the change in PaCO_2 _associated with LRM and PaCO_2 _at 10 cmH2O PEEP before an LRM; ΔPaO_2_/PaO_2 _10PEEP_noLRM_, the ratio between the change in PaO_2 _associated with LRM and PaO_2 _at 10 cmH_2_O PEEP before an LRM.

The correlations (*R*^2^) between MH/TLC (*x*) and EELV, Crs and PaCO_2 _(*y*) were 0.55, 0.57 and 0.36, respectively (*P *< 0.05) (Figure 3). There was no correlation between MH/TLC and PaO_2 _(*R*^2 ^= 0.05, *P *< 0.26).

### Sensitivity and specificity of using MH/TLC to predict effect of lung recruitment maneuver

**Figure 4:**
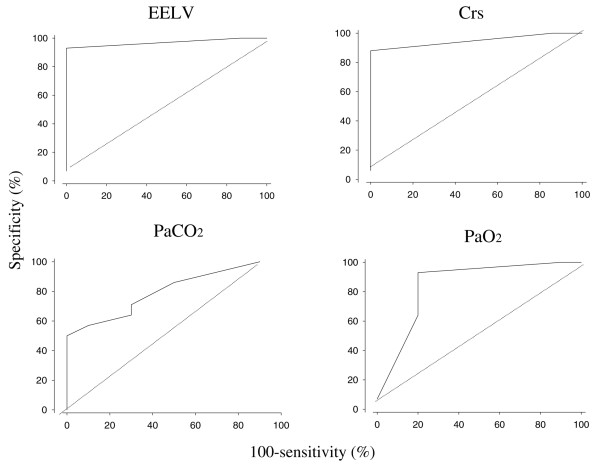
Analysis of the receiver operating characteristics curve. Analysis of the receiver operating characteristic curve (100 – sensitivity versus specificity) for the ratio between maximal volume hysteresis and total lung capacity (MH/TLC) using 40% increase in end-expiratory lung volume (EELV), 40% increase in compliance of the respiratory system (Crs), 20% decrease in partial pressure of arterial CO_2 _(PaCO_2_) and 30% increase in partial pressure of arterial oxygen (PaO_2_). See the text for explanation.

The upper (75th) centiles for the relative change by an LRM at baseline, namely before lung lavage, were 40%, 40% and 30% for EELV, Crs and PaO_2_, respectively, and the lower (25th) centile for PaCO_2 _was – 20%. These values were used in the construction of receiver operating characteristics curves for the individual measures (Figure 4). The upper angle, indicating the optimal sensitivity in relation to specificity, was found for all measures at a MH/TLC ratio of 0.3, which was used in the calculations of sensitivity and specificity. A MH/TLC ratio of more than 0.3 indicates, with a sensitivity of 1.0 and a specificity of 0.85, an improvement in EELV by an LRM. Corresponding values for Crs were 0.88 and 1.0, for PaCO_2 _0.78 and 0.60, and for PaO_2 _1.0 and 0.69.

## Discussion

The main finding in this study is that specific information from a PV loop could predict the potential for lung recruitment in a porcine model of acute lung injury.

The PV loop and lung volume measurement methods have been evaluated previously and are found to be reliable [17-19]. The short time of the PV loop procedure makes it improbable that gas exchange had a major impact of the shape of the PV loop. To obtain different lung conditions to test our hypothesis we used three models: normal lung, lung collapse, and mechanical lung injury. We used a maximal pressure of 40 cmH_2_O for the PV loops in all lung conditions to permit easy comparison of the different loops. Furthermore, 40 cmH_2_O is commonly considered safe and it would create a transpulmonary pressure high enough for obtaining an accurate TLC under the lung conditions studied. The PV loops and EELV obtained agree with previous findings: the normal lung has a crescent PV loop and the collapsed and the mechanical injured lung have a convex PV loop with reduced EELV [21,22]. In the present study, the more pronounced the convexity, as indicated by a larger MH/TLC ratio, the higher was the probability for improvements in EELV, Crs and PaCO_2 _by an LRM. This agrees well with theoretical considerations by Hickling and by Jonson and Svantesson [15,16]. Unexpectedly, although the shape of the PV loop was different from that in the injured lungs, in the normal lungs the hysteresis was substantial, with a MH/TLC ratio up to 0.3. Because the hysteresis of the PV loop at 10 cmH_2_O was equal to the increase in EELV by the LRM at similar airway pressure it could be debated whether the hysteresis found in the normal lungs was a sign of lung recruitment produced by the PV loop maneuver and thus predicted the recruitment of collapsed lung tissue. We do not believe this is the main explanation, because only minor changes were found in Crs, PaO_2 _and PaCO_2 _by the LRM. In fact, PaCO_2 _increased in four of the animals. Instead, we suggest that the probable cause was that the pressure used in the PV loop maneuver and in the LRM squeezed blood out from the lungs that was replaced by an increased amount of air in previously open lung units [23].

We used 10 cmH_2_O PEEP for two reasons: first, it is a clinically relevant PEEP level in ALI/ARDS, and second, if higher PEEP levels had been used, the inspiratory pressures would presumably have been high enough to allow tidal lung recruitment. Theoretically, tidal recruitment could inadvertently have increased EELV before LRM, because tidal recruitment might not always be followed by tidal derecruitment. This is because the PEEP used might prevent derecruitment and because the time constant for derecruitment in the lavage model is substantial [24]. In our study the inspiratory pressures were less than 22 cmH_2_O, which is well below the airway pressure needed to recruit collapsed lung parenchyma [3]. Our finding that EELV at 10 cmH_2_O before LRM was similar to the lung volume registered from the inspiratory PV loop at the same airway pressure indicates that tidal recruitment was minimal. After the LRM, EELV as measured at 10 cmH_2_O PEEP increased in all animals to similar lung volumes, as registered from the expiratory limb of the PV loop. Thus, in agreement with the findings by Vieillard-Baron and coworkers, the PV loop seems to predict the volume gain that could be achieved by an LRM [14]. However, because recruitment is dependent on time and pressure, the PV loop might not always predict the full volume effect of an LRM.

Clinically, improvement in oxygenation is often used for evaluating the effect of LRM, and it has been suggested to indicate whether recruitment of collapsed regions has occurred [10]. However, oxygenation could be improved and shunt could be decreased by a reduction in cardiac output induced by the high intrathoracic pressure during the LRM and by high PEEP [25]. It should be noted that improvements in lung mechanics or in EELV by an LRM do not necessarily indicate improvements in oxygenation, intrapulmonary shunt or CO_2 _elimination [26]. In our study, although MH/TLC was related to changes in Crs and EELV we could not find any relation to changes in PaO_2_, and the sensitivity and specificity were lower for PaO_2 _and PaCO_2 _than for Crs and EELV. However, a low MH/TLC ratio suggested that LRM would not markedly improve oxygenation, PaCO_2_, lung mechanics or EELV.

We are not aware that any simple methods have previously been reported to predict whether LRM would be effective in ALI/ARDS. The other simple clinical methods using a combination of changes in Crs, PaO_2 _and PCO_2_, or in EELV, do only evaluate *a posteriori *whether an LRM combined with high PEEP has been effective [13].

We believe that this method, using measurement of EELV combined with a PV loop, might be found valuable clinically. Registration of PV loops obtained by slowly increasing and decreasing airway pressures as well as EELV measurement methods have been incorporated in modern ventilators. Thus, in patients with low Crs and low PaO_2_/FiO_2 _ratios, EELV measurements could determine whether lung volume is reduced. Then an analysis of the shape of a PV loop could be used to predict whether an LRM and increased PEEP would be effective. Although this concept needs to be tested in patients, both the method described by Vieillard-Baron and coworkers and the method using superimposed inspiratory PV curves from different PEEP levels are conceptually similar to the method used in this study and have been found to give reliable results in patients with ARDS [11,12,14,27].

Our study has several limitations. First, it is an experiment in young previously healthy animals. Second, the lung collapse and lung injury are induced by surfactant deficiency and mechanical stress and not, as in ALI/ARDS, by local or systemic inflammation. Thus, the models used do not capture all aspects of the human disease. Third, we did not use an imaging method such as computed tomography to assess lung recruitment. Fourth, the statistics used could be criticized because the changes in EELV or lung mechanics caused by the collapse and mechanical lung injury are not independent. However, previous studies with similar models have been consistent, and therefore *a priori *we decided to use a limited number of animals.

## Conclusion

In this porcine model, specific information from a PV loop, namely a MH/TLC of 0.3, predicted better whether an LRM would improve EELV and Crs – that is, lung mechanics – than PaCO_2 _and PaO_2 _– that is, gas exchange – in the range of the studied PEEP and PV loop.

## Key messages

• Registering airway pressure – lung volume loops and measurements of end-expiratory lung volume are easily obtained at the bedside with modern ventilators.

• This animal study indicates that these measures might predict whether a lung recruitment maneuver would be effective in the treatment of acute lung injury.

## Abbreviations

ALI = acute lung injury; ARDS = acute respiratory distress syndrome; Crs = compliance of the respiratory system; ΔEELV = increase in end-expiratory lung volume at 10 cmH_2_O positive end-expiratory pressure associated with a lung recruitment maneuver; Δ*V *= difference in lung volume at 10 cmH_2_O airway pressure between the expiratory and inspiratory limbs of a static airway pressure – lung volume loop; EELV = end-expiratory lung volume; EELV-10_LRM _= end-expiratory lung volume at 10 cmH_2_O positive end-expiratory pressure after a lung recruitment maneuver; EELV-10_noLRM _= end-expiratory lung volume at 10 cmH_2_O positive end-expiratory pressure before a lung recruitment maneuver; EELV_ZEEP _= end-expiratory lung volume at zero end-expiratory pressure; ELV-10 = the absolute lung volumes at an airway pressure of 10 cmH_2_O obtained from the expiratory limb of a static airway pressure – lung volume loop; ILV-10 = the absolute lung volumes at an airway pressure of 10 cmH_2_O obtained from the inspiratory limb of an airway pressure – lung volume loop; i.m. = intramuscularly; i.v. = intravenously; MH = maximal volume hysteresis obtained from an airway pressure – lung volume loop; LRM = lung recruitment maneuver; PaCO_2 _= partial pressure of arterial CO_2_; PaO_2 _= partial pressure of arterial oxygen; PEEP = positive end-expiratory pressure; PV loop = static airway pressure – lung volume loop; TLC = total lung capacity; ZEEP = zero end-expiratory pressure.

## Competing interests

The authors declare that they have no competing interests.

## Authors' contributions

JKN participated in the design, performed the study and drafted the manuscript. NDN and AJK participated in the acquisition of the data for the study. AL participated in the design of the study, participated in the acquisition of data and helped to draft the manuscript. All authors read and approved the final manuscript.
